# Sustainable Utilization of Food Biowaste (Papaya Peel) Extract for Gold Nanoparticle Biosynthesis and Investigation of Its Multi-Functional Potentials

**DOI:** 10.3390/antiox13050581

**Published:** 2024-05-09

**Authors:** Jayanta Kumar Patra, Han-Seung Shin, In-Jun Yang, Ly Thi Huong Nguyen, Gitishree Das

**Affiliations:** 1 Research Institute of Integrative Life Sciences, Dongguk University-Seoul, Goyangsi 10326, Republic of Korea; jkpatra@dongguk.edu; 2 Department of Food Science and Biotechnology, Dongguk University-Seoul, Goyangsi 10326, Republic of Korea; spartan@dongguk.edu; 3 Department of Physiology, College of Korean Medicine, Dongguk University, Gyeongju 38066, Republic of Korea; injuny@dongguk.ac.kr (I.-J.Y.); lynguyen@uab.edu (L.T.H.N.); 4 Department of Pathology, University of Alabama at Birmingham, Birmingham, AL 35294, USA

**Keywords:** food biowaste, gold nanoparticles, antioxidant, antidiabetic, antityrosinase, anti-inflammatory, antibacterial, photocatalytic dye degradation

## Abstract

Papaya contains high amounts of vitamins A, C, riboflavin, thiamine, niacin, ascorbic acid, potassium, and carotenoids. It is confirmed by several studies that all food waste parts such as the fruit peels, seeds, and leaves of papaya are potential sources of phenolic compounds, particularly in the peel. Considering the presence of numerous bioactive compounds in papaya fruit peels, the current study reports a rapid, cheap, and environmentally friendly method for the production of gold nanoparticles (AuNPs) employing food biowaste (vegetable papaya peel extract (VPPE)) and investigated its antioxidant, antidiabetic, tyrosinase inhibition, anti-inflammatory, antibacterial, and photocatalytic degradation potentials. The phytochemical analysis gave positive results for tannins, saponins, steroids, cardiac steroidal glycoside, protein, and carbohydrates. The manufactured VPPE-AuNPs were studied by UV–Vis scan (with surface plasmon resonance of 552 nm), X-ray diffraction analysis (XRD) (with average crystallite size of 44.41 nm as per the Scherrer equation), scanning electron microscopy–energy-dispersive X-ray (SEM-EDS), thermogravimetric analysis (TGA), Fourier transform infrared spectroscopy (FT-IR), particle size, zeta potential, etc. The mean dimension of the manufactured VPPE-AuNPs is 112.2 d.nm (PDI—0.149) with a −26.1 mV zeta potential. The VPPE-AuNPs displayed a significant antioxidant effect (93.24% DPPH scavenging and 74.23% SOD inhibition at 100 µg/mL); moderate tyrosinase effect (with 30.76%); and substantial α-glucosidase (95.63%) and α-amylase effect (50.66%) at 100 µg/mL. Additionally, it was found to be very proficient in the removal of harmful methyl orange and methylene blue dyes with degradation of 34.70% at 3 h and 24.39% at 5 h, respectively. Taken altogether, the VPPE-AuNPs have been proven to possess multiple biopotential activities, which can be explored by the food, cosmetics, and biomedical industries.

## 1. Introduction

Nanotechnology has proven to have an important role in the field of technologies, biomedical science, chemical industry, drug-gene delivery, catalysis, nonlinear optical devices, photo-electrochemical applications, space industries, energy science, etc. that utilize nanoparticles or nanoscale structures. Because of their very small size which is in the nanometer range and their large surface-to-volume ratio, nanoparticles have received much attention [1,2]. Regarding novel metal nanoparticles, researchers are more focused on gold and silver nanoparticles due to the uniqueness of these nanoparticles in terms of their mechanical, electrical, size-dependent, and magnetic properties [3]. Nanotechnology is poised to contribute substantial value in the fields of curative diagnostics, agriculture, and pharmaceuticals [4,5]. Metals like silver, gold, selenium, and their oxides have been used as antioxidant agents, antibacterial agents, targeted drug delivery vehicles, anticancer agents, antimycotic agents, etc. Among them, due to their unique properties, Au and Ag nanoparticles are highly significant. Currently, is being undertaken dedicated to the synthesis of nanomaterials using physical, chemical, and biological or green synthesis methods [6,7]. Among these methods, the physical and chemical methods are considered unfavorable and have been replaced by the green synthesis methods due to the physical and chemical methods requiring multi-faceted equipment and synthesis settings and using harmful chemicals, consuming large amounts of energy, and releasing toxic chemicals with harmful byproducts [7,8]. Meanwhile, in contrast to these, the green synthesis methods involve a one-pot synthesis method utilizing minimal basic equipment and are considered non-toxic, low cost, and environmentally friendly [9,10,11].

Diverse plant parts for the synthesis of metallic nanoparticles are commonly employed in green synthesis methods for their easy availability, environmental friendliness, and lower manufacturing budget. Further, it must be noted that several plants have essential biomedical uses; they have naturally bioactive compounds, including saponins, terpenoids, flavonoids, polyphenols, alkaloids, etc. At this point, it can be suggested that if plants with essential biomedical potential are used for making metal nanoparticles (NPs), the achieved nanoparticles might be coated with the naturally active constituents and thus take on their biomedical potentials. *C. papaya* is a globally important fruit [12]. While papaya (mainly its fruit) can be eaten fresh, it is acknowledged to be generally used in food manufacturing industries to make processed foods (mainly the fruit pulp), while other parts such as the peel and seeds are discarded [13]. Some earlier reports have reported that around 32% of non-edible pulp, 8.47% of peel, and 6.51% of seeds are produced as waste during industrial food processing which are mostly discarded as food waste materials [13,14,15,16]. The accumulation of huge amounts of papaya peels (food waste) after its pulp is utilized remains an environmental issue, as the solid wastes transmit life-threatening diseases and there are no sustainable management systems for this huge and year-round production of bioresource waste materials. Thus, the use of papaya food waste materials such as peels in a sustainable manner can add to decreasing food insecurity and enhance food sustainability, specifically in the underdeveloped countries of the world [16]. There is a report of ripened papaya peels used as biofertilizers [17].

Papaya contains high amounts of vitamins A, C, riboflavin, thiamine, niacin, ascorbic acid, carotenoid, and polyphenols [13,18,19,20]. In a study by Canini et al. [21], the authors identified and quantified many bioactive compounds such as 5,7-dimethoxycoumarin, caffeic acid, p-coumaric acid, protocatechuic acid, chlorogenic acid, kaempferol, and quercetin from the leaf of the papaya plant. Azarkan et al. [22] have reported the presence of cysteine endopeptidases, chitinase, and glutaminyl cyclase in the latex of papaya. Winterhalter et al. [23] reported the existence of linalool in the fruit pulp. Olafsdottir et al. [24] reported benzylglucosinolate and benzylisothiocyanate in all tissues. Just recently, Zhou et al. [25] have studied the existence of phenolic compounds in the peel, pulp, and seeds of papaya fruits and confirmed that all these parts are potential sources of phenolic compounds, particularly in the peel. A detailed list of phenolic compounds from different parts of the papaya samples is provided in Table 4 of the article by Zhou et al. [25]. In addition, several authors have extracted compounds like isothiocyanate, chymopapain, papain, polyphenols, flavonoids, lycopene, anthocyanins, omega-3 and omega-6 fatty acids, tannins, minerals, β-carotene, etc. from papaya fruit peels [13,26,27,28,29,30,31,32]. The leading flavonoid compounds present in the papaya food waste materials (like peel, pulp, and leaves) were reported as apigenin, bemyricetin, kaempferol, quercetin, luteolin, and morin [33].

The occurrence of such bioactive compounds could somewhat clarify the pharmacological properties of this plant. Among them, papain has been reported to have a potential application in the pharmaceutical industries in the formulation of medicines and vaccines for treating several diseases associated with the digestive tract, wound treatment, and fever and vaccines for deworming cattle [13,34]. The peel extract was reported to act as a wonderful source of riboflavin, which supports the formation of bound co-enzymes such as flavin mononucleotide and flavin adenine dinucleotide, which acts as a catalyst for several reduction and oxidation reactions [30]. There are also reports that the unused parts (food waste) of papaya (peel and seeds) are rich in phenolic compounds, and flavonoids with stronger antioxidant properties compared to the flesh of the fruit [13,19,35]. Moreover, in terms of the significance of the edible part of the fruit, the chemical configuration of their byproducts is also vital not only regarding the abundance of bioactive compounds but also for the reprocessing of these food biowaste materials for several manufacturing areas, like the food, pharmaceutical, and cosmetics sectors [36,37]. Some reports show the use of papaya peels, seeds, and other parts in the treatment of stomach pains, inflammations, and bacterial infections [38], as an antioxidant, anticancer, antimicrobial, and hepatoprotective agent [39,40,41,42]. All these components present in papaya peels could be utilized as dietary and nutraceutical supplements in unique food and pharmaceutical products [43]. Because of this, currently, an effort is being made to produce AuNPs using the green vegetable *Carica papaya* peel extract as a reduction mediator and explore its multi-functional capacities in terms of antioxidant, tyrosinase inhibition, anti-inflammatory, antidiabetic, bacterial inhibition, and photocatalytic degradation potential.

## 2. Materials and Methods

### 2.1. C. papaya Extract Preparation

The *C. papaya* fruit was obtained from the nearest certified outlet of the Republic of Korea. It was identified in the laboratory and the herbarium number (RIILSEH No. 202211-04) was recorded. The waste peel of raw vegetable papaya (VPP) was washed properly, patted dry, cut into small pieces, dried in room temperature, and ground. Then, 100 g of grounded VPP was put in a 1 L flask with 500 mL double distilled water. It was heated for around 30 min and cooled down to room temperature, filtered using filter paper (Whatman No.1), and kept at 4 °C until further use.

### 2.2. Phytochemical Analysis of the VPP Extract

The extract of VPP was screened for the presence of various phytochemicals like flavonoids, saponins, tannins, terpenoids, a cardiac steroidal glycoside, carbohydrates, and anthraquinones as per the standard protocols [44,45]. For flavonoids, about 1 mL of the VPP extract was reacted with 1 mL of NaOH in a test tube, and the appearance of a dark precipitate confirmed the presence of flavonoids in the VPP extract. For saponins, VPP extract was dissolved in 10 mL of distilled water and shaken vigorously to cause frothing followed by the addition of a few drops of olive oil and checked for the appearance of foam, which was stable for around 5 min. Terpenoids were tested by chloroform assay. Briefly, 1 mL of chloroform was mixed with 2.5 mL of VPP extract and evaporated in a water bath followed by the addition of 3 mL conc. H_2_SO_4_ in boiling conditions. The formation of grey coloration confirmed the presence of terpenoids in the VPP extract. For the test of cardiac steroidal glycoside, the Keller–Kiliani test was performed. Briefly, 5 mL of VPP extract was mixed with 2 mL of glacial acetic acid and 1 drop of 2% FeCl_3_ was added followed by the addition of 0.5 mL of conc. H_2_SO_4_. The formation of a brown ring at the junction of two liquids confirmed the presence of cardiac glycoside. The Molisch reagent test was performed to check for the presence of carbohydrates, which was confirmed by the formation of purple-red rings. For testing anthraquinones, a benzene test was performed. Briefly, 3 g of VPP powder was mixed with 5 mL of benzene, soaked, and filtered followed by the addition of 5 mL of ammonia solution continuously. The appearance of red, pink, or violet coloration at the junction of two layers confirmed the presence of anthraquinones.

### 2.3. Green Synthesis and Characterization of Synthesized VPPE-AuNPs

The phytochemical-rich VPP extract was utilized for the synthesis of VPPE-AuNPs using regular procedures [46]. The characterization of the fabricated VPPE-AuNPs was performed by nine regular investigative approaches following standard methods described in detail in a previous publication [47]. UV–Vis spectral analysis was carried out by determining the scanning spectra through a UV–Vis spectrophotometer from 300 nm to 700 nm (for 24 h at regular time intervals). The alteration in the color of the test solution was recorded by visual observation. The synthesized VPPE-AuNPs’ morphology, size, and elemental compositions were scrutinized by scanning electron microscopy (SEM) and an energy-dispersive X-ray (EDS) machine linked with the SEM instrument. The morphology and surface of the synthesized VPPE-AuNPs were examined by using atomic force microscopy (AFM). The synthesized VPPE-AuNPs’ crystal structure was estimated through X-ray diffraction (XRD) analysis employing Cu-Kα radiation at 40 mA and 30 kV at a two-theta angle. The Fourier transform infrared spectroscopy (FT-IR) analysis of VPPE and VPPE-AuNPs was determined by an FT-IR spectrophotometer (Nicolet iS5 FTIR Spectrometer, Thermo-Fisher Scientific, Waltham, MA, USA) in the range of 400–4000 cm^−1^. The presence of various functional groups of compounds was determined by various modes of vibration. The zeta potential and particle size of the fabricated VPPE-AuNPs were estimated by a zeta potential machine at 25 °C. The composition and thermal stability of the synthesized VPPE-AuNPs were estimated by a thermogravimetric analysis (TGA) instrument at a temperature range of 25–900 °C with a ramping time of ten degrees Celsius per minute.

### 2.4. Assessment of Multiple Biological Potential of the Synthesized VPPE-AuNPs

The multi-functional potential of the produced VPPE-AuNPs was studied by antioxidant, antityrosinase, α-glucosidase and α-amylase inhibition, anti-inflammatory, antibacterial, and photocatalytic degradation potential studies using established standard protocols.

#### 2.4.1. Evaluation of Antioxidant Potential of VPPE-AuNPs

Evaluation of the antioxidant effect of generated VPPE-AuNPs was carried out by some assays as discussed in detail in a previous publication [47] and other standard procedures [48]. For all the antioxidant experiments, three different concentrations, i.e., 25, 50, and 100 μg/mL, of VPPE-AuNPs and gallic acid/butylated hydroxytoluene taken as reference compounds were used. To evaluate the DPPH scavenging potential of VPPE-AuNPs, gallic acid (GA) was taken as control. The absorbance was recorded at 517 nm in a spectrophotometer and the scavenging potential was evaluated through the calculation below:

(1) $$\%\;DPPH\;scavenging\;(free\;radical)\;effect=\frac{C_{trt}-T_{trt}}{C_{trt}}\times 100$$

Here, C_trt_ is the value of the control and T_trt_ is the resultant value of the treatment tested.

The ABTS scavenging effect of VPPE-AuNPs and the standard GA was estimated by a regular process [49]. Before starting the research investigation, 7.4 mM of the ABTS stock solutions and potassium persulfate 2.6 mM were prepared and mixed equally. The data were recorded at 734 nm in a spectrophotometer. The ABTS scavenging percentage was measured by Formula (1). The reducing power of VPPE-AuNPs was measured using Patra et al.’s [50] method. The OD was determined, and the data were considered as absorbance values. The SOD enzyme inhibition effect of the VPPE-AuNPs was measured by a commercial kit (Oxi Select Superoxide Dismutase Activity Assay Kit, MyBiosource, Inc., San Diego, CA, USA) by referring to the manufacturer’s standard procedure. Butylated hydroxyl toluene (BHT) was selected as control and VPPE-AuNPs were taken at concentrations of 25, 50, and 100 µg/mL. To produce superoxide anions, the assay kit uses the xanthine oxidase system, and the included chromagen generates a dye soluble in water (formazan dye) when reacting with the superoxide anions. By the inhibition of chromagen reduction, the SOD inhibition action is estimated. At 490 nm, the absorbance was recorded. The SOD inhibition percentage was estimated by following Formula (1). Further, the total antioxidant capacity (TAC) of VPPE-AuNPs is estimated by the MAK334 kit (Sigma-Aldrich, St. Louis, MO, USA) using the company’s protocol. The absorbance values were recorded at 570 nm followed by the calculation of the TAC values using the equation below.

$$TAC\;value=\frac{T_{a}{-C}_{a}}{Slope}\times n$$

where T_a_—OD of the treatment; C_a_—OD of the control; n—dilution number; slope value—standard curve linear equation.

#### 2.4.2. Evaluation of Tyrosinase Inhibitory Potential of VPPE-AuNPs

The tyrosinase inhibitory activity of VPPE-AuNPs was analyzed using the procedure of Ekennia et al. with minor amendments [51]. Briefly, the reaction mixture solution (300 μL) contains 25–100 μg/mL VPPE-AuNPs/kojic acid (KA, reference standard), L-DOPA (0.0001 M), phosphate buffer (0.0001 M, pH 6.5), and tyrosinase enzyme (mushroom, 50 U/mL). The test solution was rested for ½ an hour at room temperature. Next, after incubation, the OD was estimated at 475 nm. The VPPE-AuNPs’ tyrosinase inhibitory activity was articulated as a percentage of tyrosinase inhibition by using Formula (1).

#### 2.4.3. Antidiabetic Potential of VPPE-AuNPs

The VPPE-AuNPs’ α-amylase activity was estimated by using previously used standard procedures with a few modifications [52,53]. Concisely, test samples (40 µL, 10 mg/mL DMSO) were made again in 160 µL PBS (0.02 M, pH 6.9), comprising NaCl (0.0067 M) in 2 mL vials, and incubated for 5 min with 200 µL α-amylase (4 U/mL) which was prepared in double distilled water (ice cold). The test response was instigated by adding 400 µL potato starch (soluble) solution (1/2% *w*/*v*) in PBS (0.02 M, pH 6.9). Subsequently, incubation (3 min) followed by mixing 400 µL DNS color reagent and heating for about 10 min at 85–90 °C (water) were performed to develop color and then the mixture was cooled. In a microplate (96 wells), the test reaction solution (50 µL) was diluted with 175 µL pure water. The OD value is measured by using a spectrophotometer at 540 nm and the α-amylase effect was determined by Equation (1). To obtain an accurate OD, the background was taken according to the test sample blanks.

The α-glucosidase inhibition activity of VPPE-AuNPs was studied by following a regular process with slight modifications [53]. In the test solution (a total of 1000 µL), 4 units/mL of the α-glucosidase enzyme, VPPE-AuNPs at a concentration of 25, 50, 100 µg/mL, and potassium phosphate buffer (pH 6.8) were added. Next, the sample was incubated for 600 s (ambient conditions). Then, 3 mM p-nitrophenyl-α-d-glucopyranoside (100 μL) was used as the substrate. Afterward, the test solution was kept at 37 °C for incubation (20 min). Next, 0.1 M sodium carbonate solution (2000 µL) was mixed with it, the sample OD was measured (405 nm), and activity was estimated by Equation (1).

#### 2.4.4. Evaluation of Anti-Inflammatory Potential of VPPE-AuNPs

##### Cell Viability

For the cell viability study of VPPE-AuNPs, the cells (RAW264.7) were acquired from a cell bank, Korea and sub-cultured in culture medium (high-glucose DMEM, FBS 10%, and antibiotics 1%), at 37 °C. Using MTT assays, the effect of VPPE-AuNPs on the survival of RAW264.7 cells was studied. The cells (RAW264.7) were seeded in 96-well plates at a 5 × 10^4^ cells/well density. The detailed procedure as described in a previous publication was followed [47].

##### Enzyme-Linked Immunosorbent Test (ELISA)

In the ELISA, the cells (RAW264.7) were seeded in 96-well plates at a 5 × 10^5^ cells/well density. Next, after 1 day, cells were starved overnight with a medium that was free from serum. Then, cells (RAW264.7) were treated with the VPPE-AuNPs/dexamethasone (DEX, 0.1 mM) at different concentrations or, 1 h earlier, stimulated with LPS (lipopolysaccharide), 1 μg/mL for 1 day. Next, TNF-α, IL-1β, and IL-6 levels in culture were checked using commercial ELISA kits (Koma Biotech Inc., Seoul, Republic of Korea) using the company’s directions. The optical density was recorded at 450 nm.

#### 2.4.5. Evaluation of Antibacterial Potential of VPPE-AuNPs

The antibacterial activity of VPPE-AuNPs was evaluated against two pathogens, *Listeria monocytogenes* (ATCC 33090) and *Pediococcus* sp., by using a standard disc diffusion method [54]. A VPPE-AuNP (0.1 mg/disc) paper disc and kanamycin (0.01 mg/disc) were tested and the result was estimated after the incubation period by measuring the zone of inhibition diameter. The minimum inhibitory concentrations (MICs) of the VPPE-AuNPs were estimated by a regular technique [55]. Before starting the experiment, different concentrations of the VPPE-AuNPs were made by carrying out a two-fold dilution method. The different pathogenic bacterial cultures, around 10 µL each, were added to them separately and kept at 37 °C overnight for incubation. The MIC was estimated by naked eye observation after incubation. The lowest VPPE-AuNP concentration which did not show any noticeable development of the pathogenic bacteria visually on the nutrient broth was considered the MIC of the tested sample. The concentration of the VPPE-AuNPs which displayed no growth on nutrient agar (NA) plates was considered the minimum bactericidal concentration (MBC) of the synthesized VPPE-AuNPs.

#### 2.4.6. Evaluation of Photocatalytic Dye Degradation Potential of VPPE-AuNPs

The photocatalytic effect of the synthesized VPPE-AuNPs was estimated by degrading methylene blue (MB) and methyl orange (MO) dyes under light incubation following standard procedures with a few modifications [56]. A light treatment approach was applied to analyze the catalytic degradation ability of VPPE-AuNPs on methyl orange and methylene blue dyes. In brief, 5 ppm of a dye was added with 500 µL of VPPE-AuNPs and incubated under light exposure. To determine the dye concentrations based on the absorption spectra, 100 µL of the reaction mixture was taken out at different time intervals and scanned using a spectroscopy machine in the 300–950 nm range. The percentage of degradation was estimated employing the calculation below.

$$MB\;or\;MO\;dye\;\%\;of\;degradation=\frac{\left({Cons}_{ble}-{Cons}_{ale}\right)}{{Cons}_{ble}}\times 100$$

where ${Cons}_{ble}$ signifies the OD of the sample before light exposure and ${Cons}_{ale}$ denotes the OD of the sample after light exposure.

### 2.5. Statistical Analysis

SPSS statistical analysis software (IBM SPSS Statistics, version 27) was used for one-way ANOVA and Duncan’s multiple tests. Outcome results are displayed as means ± SD and *p*-value < 0.05. For the anti-inflammatory assay, a Student’s *t*-test for unpaired experiments was performed. OriginPro 2024 version 10.1, USA software was used for analysis and calculations.

## 3. Results and Discussion

### 3.1. VPPE-AuNP Manufacture and Analysis

**Figure 1:**
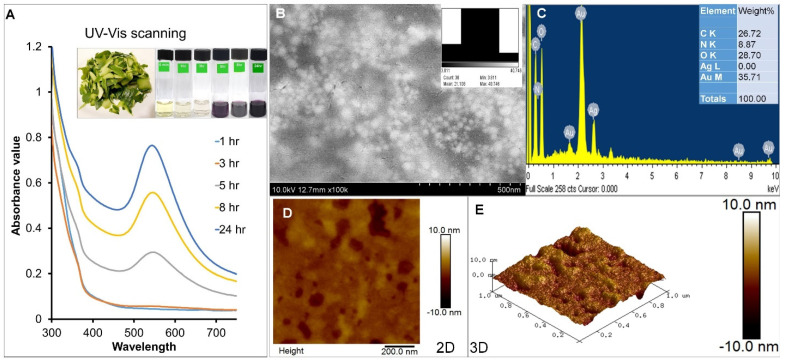
(**A**) UV–Vis spectroscopy of VPPE-AuNPs (Inset: papaya outer peels, change in color of the test solution with time); (**B**) SEM image of VPPE-AuNPs; (**C**) EDS image of VPPE-AuNPs; AFM images of VPPE-AuNPs: (**D**) 2D image; (**E**) 3D image.

At the beginning of the current research, primary screening of phytochemicals in VPP extract was performed. The phytochemical analysis gave positive results for tannins, saponins, steroids, cardiac steroidal glycoside, protein, and carbohydrates in the aqueous VPPE (Table 1). Similar results on the presence of tannins, saponins, steroids, etc. in the aqueous peel extracts were also reported previously [57,58], and our current results prove them (Table 1). It is reported that saponins have numerous beneficial effects, including the reduction of blood cholesterol and anticancer activities [59], and the presence of tannins showed that the extract is rich in polyphenolic compounds that could be responsible for its antioxidant potential [58]. In addition, the FT-IR analysis of VPPE showing the characteristic absorption bands adjacent to alcohols, phenols, and other aromatic compounds also confirmed this. In a separate study, other authors reported the presence of high levels of proteins, crude fibers, and carbohydrates in papaya fruit peels [19], and the same is reported in the current result (Table 1). Papaya peel extract has been reported to have riboflavin that helps to form bound co-enzymes such as flavin mononucleotide and flavin adenine dinucleotide, which act as a catalyst for several reduction and oxidation reactions [30], and hence these compounds also could have acted as capping and stabilizing agents in the biosynthesis of VPPE-AuNPs. This result states that the VPP extract is rich in many beneficial phytochemicals. The synthesis of VPPE-AuNPs was successful and confirmed by visual evaluation of the color of the test reaction (Figure 1A, inset). The change in color from neutral to dark purple has also been reported in previous reports [60,61] and this confirmed the successful synthesis of VPPE-AuNPs (Figure 1A, inset).

Next, the test reaction was also subjected to spectral analysis using a spectrophotometer at regular time intervals from 0 to 24 h (Figure 1A). The VPPE-AuNPs’ reaction kinetics was examined by UV–Vis spectral analysis from 300–700 nm and it displayed a maximum absorption at 552 nm (surface plasmon resonance, SPR) (Figure 1A), which is similar to earlier published reports [62,63]. As specified in earlier studies [62,63], AuNPs characteristically demonstrated just a single SPR band from 500–560 nm, and similar results were also reported here. The change in color of the VPPE-AuNPs was due to the excitation of SPR of the AuNPs as evident from previous publications [64]. The UV–Vis SPR at 552 nm, which is amplified as a function of time without hampering the peak position, signifies the characteristic bioformation of AuNPs. This result further authenticates the transformation of Au^+^ to Au°. Earlier reports have also reported similar results on the biosynthesis of AuNPs using the ethanol extract of *G. elongate* [65]. The basic analysis and elemental configuration of the VPPE-AuNPs were appraised by SEM (Figure 1B) and EDS (Figure 1C) analysis. The SEM result of VPPE-AuNPs displayed spherical-shaped nanoparticles. This result is similar to that of an earlier article [66]. The EDS profile of VPPE-AuNPs showed a strong signal for the presence of Au atoms and the sharp absorption peaks between 1–3 keV signify the existence of the Au particles (Figure 1C), which corroborates earlier reports [63,66]. Additionally, the occurrence of C, N, and O in the EDS spectra of VPPE-AuNPs could be due to the VPP extract used in the biosynthesis of VPPE-AuNPs. The 2D AFM and 3D AFM analyses were carried out to study the surface morphology of VPPE-AuNPs (Figure 1D,E). Scrutiny of the AFM images showed that the size of the VPPE-AuNPs was in the order of 0–10 nm and they appeared to be spherical in shape. Similar results were also presented previously on papaya-peel-mediated silver nanoparticles [67]. The surface of the synthesized VPPE-AuNPs was more irregular than that of the AuNPs which shows that the VPPE was successfully combined with the Au element. This is similar to an earlier reported result [68].

**Figure 2:**
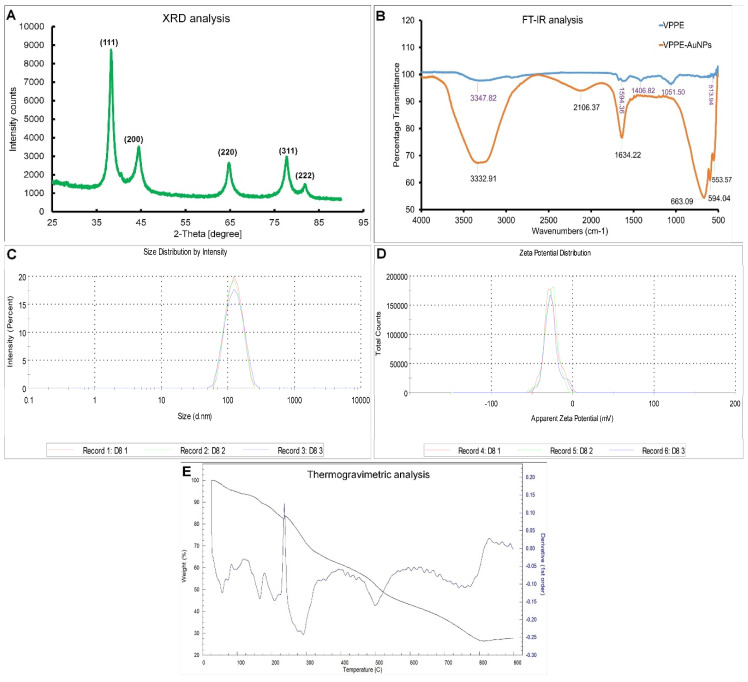
(**A**) XRD spectroscopy of VPPE-AuNPs; (**B**) FT-IR spectra of VPPE-AuNPs; (**C**) Size distribution of VPPE-AuNPs; (**D**) Zeta potential of VPPE-AuNPs; (**E**) Thermogravimetric analysis of VPPE-AuNPs.

The XRD analysis revealed that VPPE-AuNPs are crystalline in nature (Figure 2A). The Bragg’s reflections attained from the synthesized VPPE-AuNPs evidently correspond to the face-centered cubic (fcc) crystalline structure of Au. Five individual diffraction peaks ((111), (200), (220), (311), and (222)) were observed at 2θ = 38.31, 44.60, 64.80, 77.88, and 81.97 of a gold metal (JCPDS card no. 04-0783), showing the existence of pure crystalline gold [63]. The average crystallite size as per the Scherrer equation was calculated as 44.41 nm [69]. The percentage of crystallinity as calculated from the graph using the OriginPro 2024 software was found to be 86.94%.

The FT-IR analysis was undertaken to determine the type of functional groups present in the VPP extract that played a vital role in reducing and capping the VPPE-AuNPs. Five peaks at 3347.82 cm^−1^ (O–H stretching, H bonding of alcohols and phenols), 1594.36 cm^−1^ (N–H bending of primary amines), 1406.82 cm^−1^ (C–C stretching (in ring) of aromatic compounds), 1051.50 cm^−1^ (C–N stretching of aliphatic amines), and 513.94 cm^−1^ (C–Br stretching of alkyl halides) were shown by the VPP extract (Figure 2B). And six peaks at 3332.91 cm^−1^ (O-H stretching/H bonding due to alcohol and phenol groups), 2106.37 cm^−1^ (–C≡C– stretching for alkynes), 1634.22 cm^−1^ (N–H bending, bonding of primary amines), 663.09 cm^−1^, 594.04 cm^−1^, and 553.57 cm^−1^ (C-Br stretching for alkyl halides) were displayed by the VPPE-AuNPs (Figure 2B) [70]. The peak value at 1594.36 cm^−1^ (in VPPE) which was shifted to 1634.22 cm^−1^ (in VPPE-AuNPs), specifies the N–H bending and bonding of primary amine [70]. A similar peak at 1635 cm^−1^ was also reported by Aina et al. [71] when studying the synthesis of silver nanoparticles using papaya seed aqueous extract. The presence of primary amines signifying the protein content in papaya peels further evidenced that protein in the fruit’s peel could have acted as a capping and stabilizing agent in the biosynthesis of VPPE-AuNPs [72,73]. The VPP extract peaks at 3347.82, 1594.36, and 513.94 cm^−1^ probably shifted to 3332.91, 1634.22, and 594.04 cm^−1^ in VPPE-AuNPs (Figure 2B). Further, the FT-IR results signify the presence of polyphenolic compounds, flavonoids, etc. and other biomolecules in the papaya peel extract that might have acted as capping and stabilizing agents in the VPPE-AuNP synthesis process. Similar results were also reported previously [30,73,74]. The deviations in the VPP extract absorption peak values and the VPPE-AuNPs might be attributed to the VPPE-AuNP synthesis, reducing, capping, and stabilizing procedure [75].

Size distribution and zeta potential of VPPE-AuNPs were examined to determine their particle size and surface charge. The average size was detected as 112.2 d.nm, and the PDI value was 0.149 (Figure 2C). The zeta potential of the VPPE-AuNPs was found to be −26.1 mV which is highly negative (Figure 2D) and probably due to the long stability of the nanoparticles as per similar reports published previously [67,76,77]. The negative zeta potential value specifies that the negative organic molecules (such as OH^–^) are encapsulated in the NPs, which diminishes the repulsive force between the VPPE-AuNPs, hindering particle agglomeration and lengthening their stability [78]. A study by Kokila et al. [67] reported −20.5 mV zeta potential of silver nanoparticles synthesized using papaya peel extract, which is similar to the current result. The zeta average diameter size was comparatively larger than the average particle size as determined by AFM and XRD analysis because the DLS measurement is established on the hydrodynamic radius of the particles [67].

The TGA of the synthesized VPPE-AuNPs was performed to find the decomposition effect along with the thermal stability and decomposition temperature of the nanoparticles at high temperatures (25–900 °C). The TGA plot depicted a three-phase weight loss of VPPE-AuNPs from 25–900 °C (Figure 2E). The graph generated from TGA exhibited that in the first phase a total of 17.06% weight loss from 25–230 °C was attained (Figure 2E). This can be credited to the weight loss because of the water, organic solvents, physiosorbed and chemisorbed water molecules, and additional minor molecules coupled to the nanoparticle’s outer surface [79]. In the synthesis process of VPPE-AuNPs, a weight loss of 68.09% was observed in the second phase from 230–815 °C, which is credited to the elimination and decomposition of organic matter, which could have acted as the capping and stabilizing agents [46,79,80]. A residual mass of 27.66% was obtained, which might be due to the residual metal catalysts from synthesis or impurities within the sample [81].

### 3.2. The Multi-Biofunctional Potential of VPPE-AuNPs

The VPPE-AuNPs’ multi-functional biopotential was estimated by the antioxidant, antityrosinase, antidiabetic, anti-inflammatory, antibacterial, and photocatalytic dye degradation properties.

#### 3.2.1. Antioxidant Effect of VPPE-AuNPs

**Figure 3:**
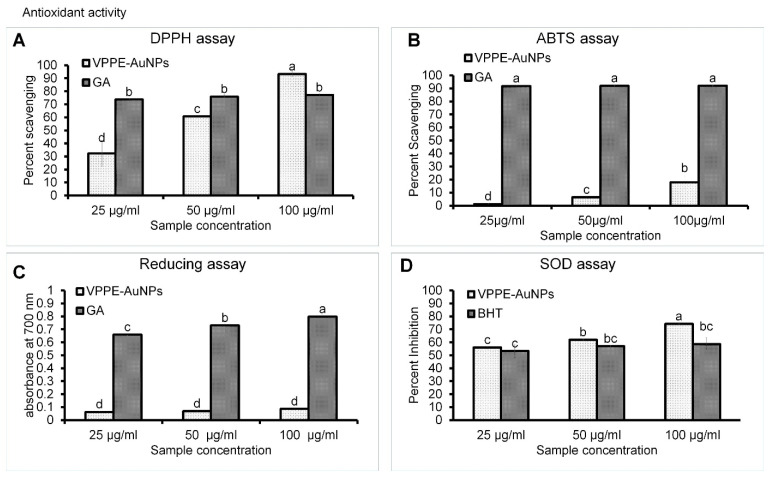
The antioxidant free radical scavenging potential of VPPE-AuNPs. (**A**): DPPH scavenging assay; (**B**): ABTS scavenging assay; (**C**): Reducing power assay; (**D**): SOD inhibition assay. Different superscript letters indicate statistical significance at *p* < 0.05.

Antioxidant compounds are highly capable of inhibiting the reactions of oxidative chains, which occur in the presence of reactive oxygen species. They are proficient in stabilizing polymeric products like petrochemicals, pharmaceuticals, foodstuffs, cosmetics, etc. [82]. These compounds play a significant part in the body’s natural resistance mechanism, as they are responsible for protection against the harmful effects of free radicals. The antioxidant effect of VPPE-AuNPs was evaluated by various in vitro analyses. The obtained results are displayed in Figure 3. The DPPH scavenging result of VPPE-AuNPs displayed a significant effect with 93.24% scavenging at 100 µg/mL in contrast to standard GA (77.19%) at the same concentration (Figure 3A). The ABTS free radical scavenging effect of VPPE-AuNPs displayed a positive value of 17.92% scavenging in contrast to GA positive standard (92.03%) scavenging at a 100 µg/mL concentration (Figure 3B). Likewise, the VPPE-AuNPs also exhibited a positive reducing power effect at three concentrations (25, 50, 100 µg/mL) that gradually amplified (Figure 3C). Moreover, the VPPE-AuNPs also displayed a high SOD inhibition effect of 74.23% (100 µg/mL) in contrast to the 58.67% inhibition effect displayed by the positive control BHT at the same concentration (Figure 3D). The VPPE-AuNPs’ scavenging potential was improved with the increased sample concentration, which may be attributed to their capability to deliver ions like electrons to the free radicals to lessen their harmful effects [83]. It is concluded that the DPPH effect of the VPPE-AuNPs is comparatively high among all four tested assays (Figure 3). In addition, the effective concentration showing 50% activity (IC_50_/IC_0.5_ values) according to all antioxidant parameters was calculated and is presented in Table 2. Concerning the IC_50_/IC_0.5_ values, the DPPH and SOD scavenging potentials of VPPE-AuNPs are highly commendable at 44.54 and 43.34 µg/mL, respectively (Table 2). The result of this study corroborates the previously reported findings by Anadozie et al. [84], where higher concentrations of their synthesized AuNPs using extract of papaya fruit exhibited high antioxidant activity. Further, the authors reported an IC_50_ of 0.60 mg/mL for DPPH activity [84], which is 10 times higher than what is shown by the VPPE-AuNPs (Table 2). It proves that the VPP-extract-mediated AuNPs are more effective compared to AuNPs made from other papaya parts. Likewise, other authors [3] reported the antioxidant potential of selenium nanoparticles synthesized using papaya fruit extract and found that the IC_50_ values for DPPH and ABTS assay were 45.65 and 43.06 µg/mL, respectively, which are similar to the current results obtained by VPPE-AuNPs. In another study, Easmin et al. [85] reported the antioxidant potential of zinc oxide nanoparticles synthesized using papaya peel extract with an IC_50_ value of 98.74 µg/mL for DPPH assay compared to 44.54 µg/mL for the IC_50_ value exhibited by the current VPPE-AuNPs. In a study by Jeon et al. [42], very little DPPH and ABTS activity of the papaya peel extract was reported, however, when AuNPs were synthesized using a peel extract (VPPE) in the current investigation, their activities were enhanced (Figure 3, Table 2). Additionally, the TACs of VPPE-AuNPs and GA were found to be 45.64 ± 1.38 and 47.48 ± 1.76 µM Trolox equivalent, respectively (Table 2). In a study by Salla et al. [86], the authors estimated the antioxidant potential of papaya fruit peel extracts and concluded that TAC was 14.56 mM Trolox equivalents and DPPH activity had an IC_50_ of 8.33 mg/mL, which is much higher than what is exhibited by the VPPE-AuNPs (Table 2). Hence, it can be assumed that when AuNPs are synthesized using VPPE as the reducing and capping agent, the antioxidant potential is increased by several fold as evident from the current results. The antioxidant potential of papaya-peel-mediated silver nanoparticles has also been reported previously [67] and this proves the current claim of promising antioxidant potential of papaya-peel-based nanoparticles. All these findings confirmed the superior antioxidant potential of the current synthesized VPPE-AuNPs.

VPPE-AuNPs were confirmed to possess a substantial amount of antioxidant effects, which may be due to the phytochemicals existing in the extract of VPP that represented capping and stabilizing agents in the nanoparticle production process. The presence of many phytochemicals including tannins in the VPPE showed that the extract is rich in polyphenolic compounds that could be responsible for its antioxidant potential as evident in the current result [58,86]. There are reports that the waste parts of papaya (peel and seeds) are rich in phenolic compounds and flavonoids with stronger antioxidant properties compared to the flesh of the fruit [19,35]. Jeon et al. [42] have quantified the individual phenolic compounds present in different waste parts (like peel, pulp, and leaves) of papaya and have reported that the leading flavonoid compounds present in papaya food waste materials (like peel, pulp, and leaves) are apigenin, bemyricetin, kaempferol, quercetin, luteolin, and morin [33,42]. Hence, the antioxidant potential of VPPE-AuNPs, which were synthesized by using outer peel papaya extract as a reducing and capping agent, could also be attributed to the presence of these compounds.

#### 3.2.2. The Antityrosinase Potential of VPPE-AuNPs

**Figure 4:**
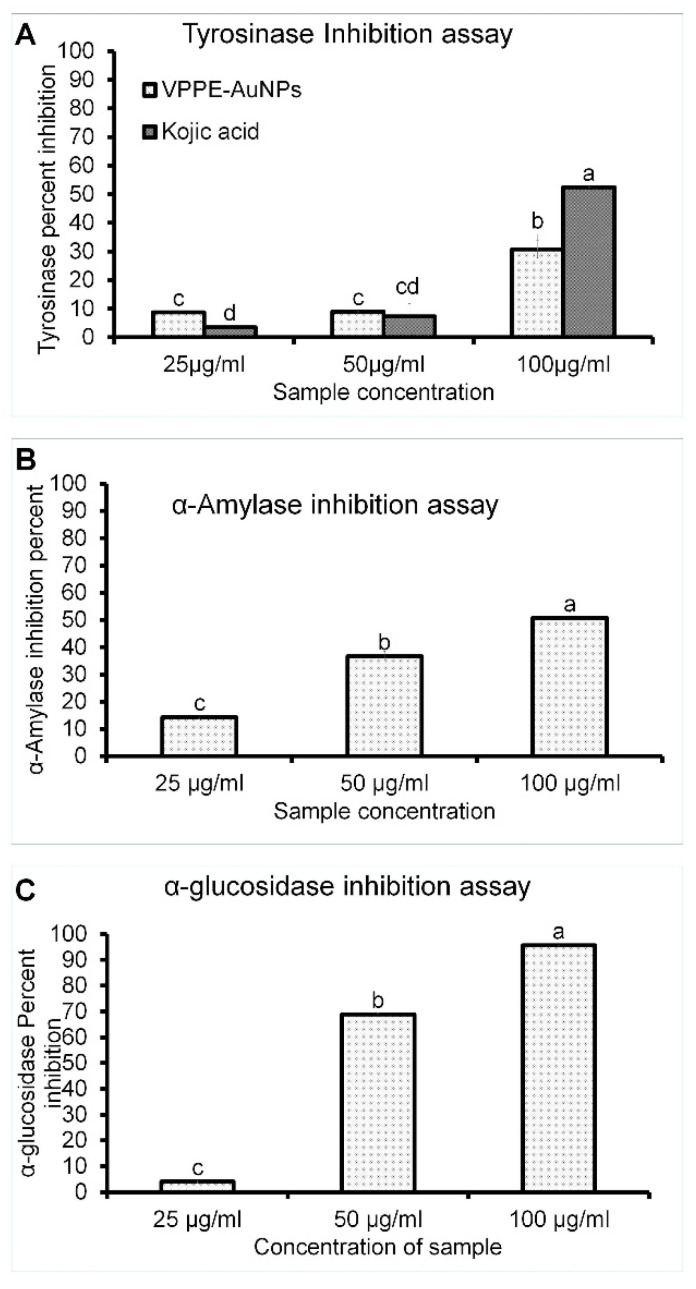
(**A**): Tyrosinase inhibitory potential; (**B**): α-amylase inhibition; and (**C**): α-glucosidase inhibition potential of VPPE-AuNPs. Different superscript letters indicate statistical significance at *p* < 0.05.

The tyrosinase enzyme assay was performed against a mushroom tyrosine enzyme to evaluate the effectiveness of VPPE-AuNPs on melanin-producing enzymes. The obtained result is presented in Figure 4A. The VPPE-AuNPs displayed a moderate tyrosinase inhibition effect of 30.76% (100 µg/mL) in contrast to KA which displayed an effect of 52.42% at an equal concentration (Figure 4A). The IC_50_ values of VPPE-AuNPs and KA were calculated as 222.04 µg/mL and 218.68 µg/mL, respectively (Table 2). Earlier reports suggest that melanin is formed by the oxidation of L-tyrosine followed by its conversion to L-dihydroxy-phenylalanine (L-DOPA) by catalysis with a copper-dependent tyrosinase enzyme [87,88]. Despite its protecting role, excess melanin production in the body can result in hyperpigmentation, melasma, and age spots [89]. Therefore, VPPE-AuNPs with positive tyrosinase inhibitory effects could be considered a useful candidate by the cosmetics industry in the production of skincare products after intensive safety testing and approval of safety guidelines.

#### 3.2.3. α-Glucosidase and α-Amylase Assay of VPPE-AuNPs

The VPPE-AuNPs displayed good α-amylase inhibition (50.66%, 100 µg/mL), with an IC_50_ value of 84.87 µg/mL (Table 2, Figure 4B) and a significantly high α-glucosidase enzyme inhibition (95.63%, 100 µg/mL, and IC_50_—44.29 µg/mL) (Table 2, Figure 4C). Both enzymes are important to minimize hyperglycemia, suppression of which will eventually decrease the breaking of carbohydrates to monosaccharides and other simpler forms which affect blood sugar levels and result in high blood sugar [90]. The treatment of diabetes can be carried out by decreasing the effects of α-glucosidase and α-amylase to slow down the release of glucose in the blood [91,92]. Therefore, the VPPE-AuNPs might be a potential candidate to treat diabetes. The antidiabetic potential of papaya leaf extract was reported previously [93] and the current findings support it.

#### 3.2.4. Anti-Inflammatory Prospects of VPPE-AuNPs

**Figure 5:**
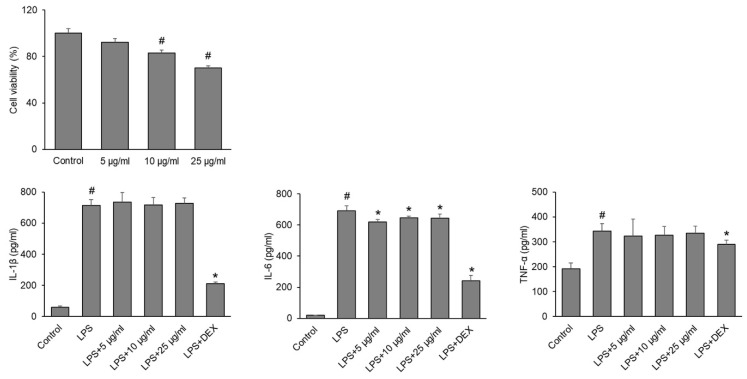
Anti-inflammatory potential of VPPE-AuNPs. ^#^ *p* < 0.05 vs. Control, * *p* < 0.05 vs. LPS.

The anti-inflammatory results indicated that the cell viability decreased at 10 and 25 µg/mL concentrations (Figure 5). However, at 10 µg/mL, the cell viability is around 80% (Figure 5). Therefore, it is concluded that the toxicity of VPPE-AuNPs is not very strong. In the inflammatory process, cytokines (proinflammatory), like IL-6, IL-1β, and TNF-α, are highly important [94]. Here, VPPE-AuNPs hinder the manufacture of proinflammatory cytokine (IL-6) increased by LPS treatment (Figure 5). Hence, VPPE-AuNPs are judged to have an anti-inflammatory effect. The result of the current research is similar to that of earlier published reports [94,95]. In a study by Jeon et al. [42], the authors demonstrated the positive anti-inflammatory potential of papaya peel extracts and this is also proved in the current study. Earlier studies have proposed the anti-inflammatory potential of papaya peel extracts and have shown that they have the ability to control the inflammatory factors in many cell types, which are exposed to different stresses [96]. Pathak et al. [97] have reported that the flavonoid-rich fraction of papaya seed extract inhibited the activation of NF-κB, as well as INF-γ, IL-6, and TNF-α, in colon, kidney, lung, and pancreatic cells. Consequently, the anti-inflammatory potential of the VPPE-AuNPs (Figure 5) could be associated with the flavonoids present in the VPPE that acted as the reducing and capping agents during the biosynthesis of the nanoparticles.

#### 3.2.5. Antibacterial Potential of VPPE-AuNPs

VPPE-AuNPs’ antibacterial effect was evaluated against two pathogenic bacteria (foodborne). The results of antibacterial activity are presented in Table 3. The VPPE-AuNPs demonstrated a positive antibacterial effect at 100 µg/disc with a 10.00 ± 0.18 mm zone of inhibition against *Pediococcus* sp. and 8.74 ± 0.03 mm against *Listeria monocytogenes* ATCC 33090 (Table 3). Kanamycin was taken as the standard positive control (10 µg/disc) and it showed a diameter of the zone of inhibition of 12.00 ± 0.05 mm against *Pediococcus* sp. and 13.04 ± 0.14 mm against *L. monocytogenes* (Table 3). The VPPE-AuNPs could be a substitute for synthetic antibacterial agents and can be explored by the cosmetics industry. In addition, after several safety tests and approvals, they can also be explored for use synergistically in combination with other antibiotics and thus can aid in tackling multidrug-resistant pathogens. Further, the MIC and MBC of the VPPE-AuNPs were found to be 100 µg/mL and ˃100 µg/mL, respectively. Kanamycin’s MIC and MBC were 5–10 µg/mL (Table 3). There are a few previous reports on the antibacterial activity of papaya-peel-mediated silver nanoparticles against pathogenic bacteria [30,98,99]. The positive antibacterial effect of zinc oxide nanoparticles synthesized using papaya peel extract has also been reported [85]. All these claims also justify the current claim of the positive antibacterial potential of VPPE-AuNPs. The reason for the antibacterial effect of VPPE-AuNPs could be hypothesized as being their smaller size, allowing them to easily enter the bacterial cells, lysing the cell wall or disturbing the cell membrane permeability, osmoregulation, and transport of electrons in the cell, leading to cell death of the bacterial pathogen [54,98,100,101]. Lesser antibacterial activity exhibited by the VPPE-AuNPs could be attributed to both the bacterial pathogens being Gram-positive bacteria, which could have hindered the entry of the NPs into the cell wall which is made up of a thicker three-dimensional peptidoglycan layer with linear polysaccharide chains cross-linked by more short peptides forming a more complex structure [67,98,102,103]. Additionally, the positively charged metal ions released from NPs might have interacted with the negatively charged bacterial surface, resulting in the dissolution and weakening of the surface protein of the bacteria, leading to cell death [79,104].

#### 3.2.6. Photocatalytic Dye Degradation Effect of VPPE-AuNPs

**Figure 6:**
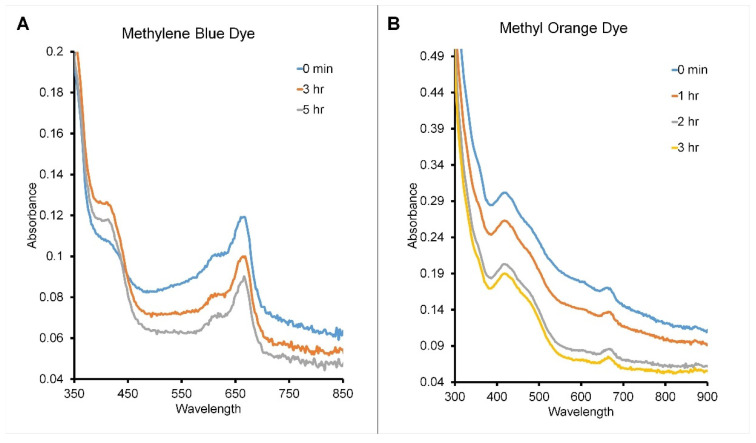
Photocatalytic degradation of (**A**) methylene blue and (**B**) methyl orange dyes by the VPPE-AuNPs.

The photocatalytic degradation effect by the VPPE-AuNPs was estimated for two types of industrial toxic dyes, methylene blue and methyl orange, under light exposure treatment (Figure 6). With the VPPE-AuNP treatment, the absorbance value for the methylene blue and methyl orange dyes decreased with the increase in light exposure time, as shown in Figure 6. The effectiveness of the VPPE-AuNPs in the case of methylene blue dye degradation was evaluated at 666 nm at different time intervals for 5 h. When the VPPE-AuNPs were added to the dye solution and exposed to light, they exhibited a time-dependent reduction in the intensity of the absorption peak value. After 5 h of light exposure (in the case of methylene blue dye), the estimated dye degradation percentage was found to be around 24.39% (Figure 6A). The effectiveness of VPPE-AuNPs in the case of the methyl orange degradation was evaluated at 418 nm at different time intervals for 3 h (Figure 6B). After 3 h of light exposure (in the case of methyl orange dye), the calculated dye degradation percentage was found to be around 34.70% (Figure 6B). It is stated that the photocatalytic effect depends on the morphology and form of metallic NPs [56,105,106]. In addition, there is also evidence that spherical nanoparticles showed a greater photocatalytic degradation effect [56,107]. The catalytic degradation of 4-nitrophenol by silver nanoparticles synthesized using papaya peel extract has been reported by Prasad et al. [74]. In another study, the photocatalytic degradation of palm oil mill effluents by copper oxide nanoparticles synthesized by papaya peel aqueous extract was also reported [73]. Similarly, Easmin et al. [85] have reported the photocatalytic degradation of rhodamine B by papaya-peel-mediated zinc oxide nanoparticles. All these studies further validated the promising photocatalytic degradation effect of VPPE-AuNPs in the current investigation. The VPPE-AuNPs could be explored as a promising candidate for light-based wastewater management.

## 4. Conclusions

Cost-effective, harmless, biofabricated VPPE-AuNPs were effectively synthesized using VPP extract. In the synthesis process of VPPE-AuNPs, the phytochemicals of VPP extract acted as initiators and stabilizers. The application of papaya waste peels in the fabrication of AuNPs is a profitable and environmentally friendly technique. In addition, it is a proficient technique for the management of waste utilization from the vegetable and food industries. To the best of our knowledge, this is the first reported research to synthesize AuNPs from green raw vegetable papaya waste peel extract and explore its biological activities like antioxidant, antidiabetic, antibacterial, antityrosinase, wound healing, anti-inflammatory, and photocatalytic degradation activities. The biofabricated VPPE-AuNPs exhibited substantial biological multi-therapeutic effects. VPPE-AuNPs also exhibited photocatalytic degradation potential against methyl orange and methylene blue dyes. Considering the above results, the useful multi-biopotential properties of the VPPE-AuNPs could be explored for applications in the cosmetics and food sector industries such as applications as sunscreen and antibacterial agents, etc., based on their promising antioxidant, α-glucosidase and α-amylase enzyme inhibition, antibacterial, anti-inflammatory, and tyrosinase inhibitory potentials. They can also be a promising candidate for light-based wastewater treatment. The above results confirm that VPPE-AuNPs have multi-potential with versatile applications. The present study represents a recent unique trend in the exclusive utilization of the vegetable waste peels of *Carica papaya* as an absolute and rich resource for biological application.

## Figures and Tables

**Table 1:** Phytochemical results of VPPE.

Name	Result
Tannin	+ve
Protein, amino acids	+ve
Saponin	+ve
Cardiac steroidal glycoside	+ve
Carbohydrates	+ve
Steroids	+ve

+ve—positive.

**Table 2:** IC_50_/IC_0.5_ values of antioxidant, total antioxidant capacity, tyrosinase inhibitory, and antidiabetic assays of VPPE-AuNPs.

Antioxidant Assays	IC_50_ Value (µg/mL)	
	VPPE-AuNPs	GA
DPPH free radical scavenging	44.54	24.96
ABTS free radical scavenging	566.39	31.71
SOD inhibition	43.34	50.86 (BHT)
Reducing power (IC_0.5_)	580.91	38.62
TAC (µM Trolox equivalent)	45.64 ± 1.38	47.48 ± 1.76
Tyrosinase inhibitory activity	222.04	218.68 (Kojic acid)
α-glucosidase inhibition	44.29	
α-amylase inhibition	84.87	

**Table 3:** VPPE-AuNP and Kanamycin’s effect on the infective strains.

Infective Strains	Measurement of Inhibition (mm)		IC and BC			
			VPPE-AuNPs		Kanamycin	
	Sample	Standard	IC	BC	IC	BC
*Pediococcus* sp.	10.00 ± 0.18	12.00 ± 0.05	100	>100	5	10
*Listeria monocytogenes* ATCC 33090	8.74 ± 0.03	13.04 ± 0.14	100	>100	5	10

Sample (VPPE-AuNPs—100 µg/disc); Standard (Kanamycin—10 µg/disc); IC—MIC (µg/mL); BC—MBC (µg/mL).

## Data Availability

The original contributions presented in the study are included in the article as figures and tables.

## References

[1] Ray P.C. (2010). Size and shape dependent second order nonlinear optical properties of nanomaterials and their application in biological and chemical sensing. Chem. Rev..

[2] Bhardwaj K., Chopra C., Bhardwaj P., Dhanjal D.S., Singh R., Najda A., Cruz-Martins N., Singh S., Sharma R., Kuča K. (2022). Biogenic Metallic Nanoparticles from Seed Extracts: Characteristics, Properties, and Applications. J. Nanomater..

[3] Vundela S.R., Kalagatur N.K., Nagaraj A., Kadirvelu K., Chandranayaka S., Kondapalli K., Hashem A., Abd_Allah E.F., Poda S. (2022). Multi-biofunctional properties of phytofabricated selenium nanoparticles from Carica papaya fruit extract: Antioxidant, antimicrobial, antimycotoxin, anticancer, and biocompatibility. Front. Microbiol..

[4] Khan S.A., Shahid S., Lee C.-S. (2020). Green synthesis of gold and silver nanoparticles using leaf extract of Clerodendrum inerme; characterization, antimicrobial, and antioxidant activities. Biomolecules.

[5] Angel R.-G.M., Francisco R.-F., Enrique M.-R., Antonio A.J., Arturo B.-R., Jacobo A.-M. (2016). Applications of Nanotechnology in The Agriculture, Food, and Pharmaceuticals. Nanosci. Nanotechnol..

[6] Ying S., Guan Z., Ofoegbu P.C., Clubb P., Rico C., He F., Hong J. (2022). Green synthesis of nanoparticles: Current developments and limitations. Environ. Technol. Innov..

[7] Horwat D., Zakharov D., Endrino J., Soldera F., Anders A., Migot S., Karoum R., Vernoux P., Pierson J. (2011). Chemistry, phase formation, and catalytic activity of thin palladium-containing oxide films synthesized by plasma-assisted physical vapor deposition. Surf. Coat. Technol..

[8] Alsammarraie F.K., Wang W., Zhou P., Mustapha A., Lin M. (2018). Green synthesis of silver nanoparticles using turmeric extracts and investigation of their antibacterial activities. Colloids Surf. B Biointerfaces.

[9] Devi H.S., Boda M.A., Shah M.A., Parveen S., Wani A.H. (2019). Green synthesis of iron oxide nanoparticles using Platanus orientalis leaf extract for antifungal activity. Green Process. Synth..

[10] Vijayaram S., Razafindralambo H., Sun Y.Z., Vasantharaj S., Ghafarifarsani H., Hoseinifar S.H., Raeeszadeh M. (2024). Applications of Green Synthesized Metal Nanoparticles—A Review. Biol. Trace Elem. Res..

[11] Singh J., Dutta T., Kim K.-H., Rawat M., Samddar P., Kumar P. (2018). ‘Green’ synthesis of metals and their oxide nanoparticles: Applications for environmental remediation. J. Nanobiotechnol..

[12] Evans E.A., Ballen F.H., Crane J.H. (2012). An overview of US papaya production, trade, and consumption. EDIS.

[13] Soares C.S.B. (2021). Papaya (*Carica papaya* L.) By-Products: Characterization and Valorisation of Bioactive and Energetic Potential. Master’s Thesis.

[14] Morais D.R., Rotta E.M., Sargi S.C., Schmidt E.M., Bonafe E.G., Eberlin M.N., Sawaya A.C., Visentainer J.V. (2015). Antioxidant activity, phenolics and UPLC–ESI (–)–MS of extracts from different tropical fruits parts and processed peels. Food Res. Int..

[15] Anwar M., Rasul M.G., Ashwath N., Nabi M.N. (2019). The potential of utilising papaya seed oil and stone fruit kernel oil as non-edible feedstock for biodiesel production in Australia—A review. Energy Rep..

[16] Vinha A.F., Costa A.S., Espírito Santo L., Ferreira D.M., Sousa C., Pinto E., Almeida A., Oliveira M.B.P. (2024). High-Value Compounds in Papaya By-Products (*Carica papaya* L. var. Formosa and Aliança): Potential Sustainable Use and Exploitation. Plants.

[17] Dahunsi S., Oranusi S., Efeovbokhan V., Adesulu-Dahunsi A., Ogunwole J. (2021). Crop performance and soil fertility improvement using organic fertilizer produced from valorization of Carica papaya fruit peel. Sci. Rep..

[18] Lydia E., John S., Mohammed R., Sivapriya T. (2016). Investigation on the Phytochemicals present in the Fruit peel of Carica papaya and evaluation of its Antioxidant and Antimicrobial property. Res. J. Pharmacogn. Phytochem..

[19] Martial-Didier A.K., Hubert K.K., Parfait K.E.J., Kablan T. (2017). Phytochemical properties and proximate composition of papaya (*Carica papaya* L. var solo 8) peels. Turk. J. Agric. Food Sci. Technol..

[20] Sancho L.E.G.-G., Yahia E.M., González-Aguilar G.A. (2011). Identification and quantification of phenols, carotenoids, and vitamin C from papaya (*Carica papaya* L., cv. Maradol) fruit determined by HPLC-DAD-MS/MS-ESI. Food Res. Int..

[21] Canini A., Alesiani D., D’Arcangelo G., Tagliatesta P. (2007). Gas chromatography–mass spectrometry analysis of phenolic compounds from *Carica papaya* L. leaf. J. Food Compos. Anal..

[22] Azarkan M., Clantin B., Bompard C., Belrhali H., Baeyens-Volant D., Looze Y., Villeret V., Wintjens R. (2005). Crystallization and preliminary X-ray diffraction studies of the glutaminyl cyclase from Carica papaya latex. Acta Crystallogr. Sect. F Struct. Biol. Cryst. Commun..

[23] Winterhalter P., Katzenberger D., Schreier P. (1986). 6,7-Epoxy-linalool and related oxygenated terpenoids from Carica papaya fruit. Phytochemistry.

[24] Olafsdottir E.S., Jørgensen L.B., Jaroszewski J.W. (2002). Cyanogenesis in glucosinolate-producing plants: Carica papaya and Carica quercifolia. Phytochemistry.

[25] Zhou Y., Cao Y., Li J., Agar O.T., Barrow C., Dunshea F., Suleria H.A. (2023). Screening and characterization of phenolic compounds by LC-ESI-QTOF-MS/MS and their antioxidant potentials in papaya fruit and their by-products activities. Food Biosci..

[26] Parniakov O., Roselló-Soto E., Barba F.J., Grimi N., Lebovka N., Vorobiev E. (2015). New approaches for the effective valorization of papaya seeds: Extraction of proteins, phenolic compounds, carbohydrates, and isothiocyanates assisted by pulsed electric energy. Food Res. Int..

[27] Thomás G.-E., Rodolfo H.-G., Juan M.-D., Georgina S.-F., Luis C.-G., Ingrid R.-B., Santiago G.-T. (2009). Proteolytic activity in enzymatic extracts from *Carica papaya* L. cv. Maradol harvest by-products. Process Biochem..

[28] Coimbra M.C., Jorge N. (2012). Fatty acids and bioactive compounds of the pulps and kernels of Brazilian palm species, guariroba (*Syagrus oleraces*), jerivá (*Syagrus romanzoffiana*) and macaúba (*Acrocomia aculeata*). J. Sci. Food Agric..

[29] Pavithra C.S., Devi S.S., Suneetha W.J., Rani C.V. (2018). Nutritional Profiling of Papaya Peel Incorporated Chapathis. Chem. Sci. Rev. Lett..

[30] Balavijayalakshmi J., Ramalakshmi V. (2017). Carica papaya peel mediated synthesis of silver nanoparticles and its antibacterial activity against human pathogens. J. Appl. Res. Technol..

[31] Dotto J.M., Abihudi S.A. (2021). Nutraceutical value of *Carica papaya*: A review. Sci. Afr..

[32] Huet J., Looze Y., Bartik K., Raussens V., Wintjens R., Boussard P. (2006). Structural characterization of the papaya cysteine proteinases at low pH. Biochem. Biophys. Res. Commun..

[33] Iordănescu O.A., Băla M., Gligor D., Zippenfening S.E., Cugerean M.I., Petroman M.I., Hădărugă D.I., Hădărugă N.G., Riviş M. (2021). A DPPH· Kinetic Approach on the Antioxidant Activity of Various Parts and Ripening Levels of Papaya (*Carica papaya* L.) Ethanolic Extracts. Plants.

[34] Yahia E.M. (2011). Postharvest Biology and Technology of Tropical and Subtropical Fruits: Mangosteen to White Sapote.

[35] Insanu M., NMDMW N., Solihin L., Wirasutisna K. (2021). Antioxidant activities and phytochemicals of polar, semi-polar, and nonpolar extracts of used and unused parts of *Carica papaya* fruit. Biocatal. Agric. Biotechnol..

[36] Yogiraj V., Goyal P.K., Chauhan C.S., Goyal A., Vyas B. (2014). *Carica papaya* Linn: An overview. Int. J. Herb. Med..

[37] Sharma A., Bachheti A., Sharma P., Bachheti R.K., Husen A. (2020). Phytochemistry, pharmacological activities, nanoparticle fabrication, commercial products and waste utilization of Carica papaya L.: A comprehensive review. Curr. Res. Biotechnol..

[38] Ghaffarilaleh V., Fisher D., Henkel R. (2019). Carica papaya seed extract slows human sperm. J. Ethnopharmacol..

[39] Zhang R., Lv J., Yu J., Xiong H., Chen P., Cao H., John Martin J.J. (2022). Antioxidant Analysis of Different Parts of Several Cultivars of Papaya (*Carica papaya* L.). Int. J. Fruit Sci..

[40] He X., Ma Y., Yi G., Wu J., Zhou L., Guo H. (2017). Chemical composition and antifungal activity of Carica papaya Linn. seed essential oil against *Candida* spp.. Lett. Appl. Microbiol..

[41] Devanesan S., Jayamala M., AlSalhi M.S., Umamaheshwari S., Ranjitsingh A.J.A. (2021). Antimicrobial and anticancer properties of Carica papaya leaves derived di-methyl flubendazole mediated silver nanoparticles. J. Infect. Public Health.

[42] Jeon Y.A., Chung S.W., Kim S.C., Lee Y.J. (2022). Comprehensive assessment of antioxidant and anti-inflammatory properties of papaya extracts. Foods.

[43] Pathak P.D., Mandavgane S.A., Kulkarni B.D. (2019). Waste to wealth: A case study of papaya peel. Waste Biomass Valorization.

[44] Gul R., Jan S.U., Faridullah S., Sherani S., Jahan N. (2017). Preliminary phytochemical screening, quantitative analysis of alkaloids, and antioxidant activity of crude plant extracts from *Ephedra intermedia* indigenous to Balochistan. Sci. World J..

[45] Sofowora A. (1993). Medicinal Plants and Medicine in Africa.

[46] Patra J.K., Baek K.-H. (2016). Comparative study of proteasome inhibitory, synergistic antibacterial, synergistic anticandidal, and antioxidant activities of gold nanoparticles biosynthesized using fruit waste materials. Int. J. Nanomed..

[47] Das G., Seo S., Yang I.-J., Nguyen L.T.H., Shin H.-S., Patra J.K. (2023). Synthesis of Biogenic Gold Nanoparticles by Using Sericin Protein from Bombyx mori Silk Cocoon and Investigation of Its Wound Healing, Antioxidant, and Antibacterial Potentials. Int. J. Nanomed..

[48] Zhou Y., Itoh H., Uemura T., Naka K., Chujo Y. (2001). Preparation of π-conjugated polymer-protected gold nanoparticles in stable colloidal form. Chem. Commun..

[49] Das G., Shin H.S., Patra J.K. (2022). Multitherapeutic Efficacy of Curly Kale Extract Fabricated Biogenic Silver Nanoparticles. Int. J. Nanomed..

[50] Patra J.K., Kim S.H., Hwang H., Choi J.W., Baek K.-H. (2015). Volatile Compounds and Antioxidant Capacity of the Bio-Oil Obtained by Pyrolysis of Japanese Red Pine (Pinus Densiflora Siebold and Zucc.). Molecules.

[51] Ekennia A., Uduagwu D., Olowu O., Nwanji O., Oje O., Daniel B., Mgbii S., Emma-Uba C. (2021). Biosynthesis of zinc oxide nanoparticles using leaf extracts of *Alchornea laxiflora* and its tyrosinase inhibition and catalytic studies. Micron.

[52] Ali H., Houghton P., Soumyanath A. (2006). α-Amylase inhibitory activity of some Malaysian plants used to treat diabetes; with particular reference to *Phyllanthus amarus*. J. Ethnopharmacol..

[53] Gowri P.M., Tiwari A.K., Ali A.Z., Rao J.M. (2007). Inhibition of α-glucosidase and amylase by bartogenic acid isolated from *Barringtonia racemosa* Roxb. seeds. Phytother. Res..

[54] Patra J.K., Baek K.-H. (2015). Novel green synthesis of gold nanoparticles using *Citrullus lanatus* rind and investigation of proteasome inhibitory activity, antibacterial, and antioxidant potential. Int. J. Nanomed..

[55] Kubo I., Fujita K.-I., Kubo A., Nihei K.-I., Ogura T. (2004). Antibacterial activity of coriander volatile compounds against *Salmonella choleraesuis*. J. Agric. Food Chem..

[56] Selvam K., Albasher G., Alamri O., Sudhakar C., Selvankumar T., Vijayalakshmi S., Vennila L. (2022). Enhanced photocatalytic activity of novel *Canthium coromandelicum* leaves based copper oxide nanoparticles for the degradation of textile dyes. Environ. Res..

[57] Roshni A., Lubaina A. (2023). Phytochemical investigation, FT-IR profiling and antibacterial activity of papaya (var. red lady) peel—A rich source of bioactive chemicals. J. Adv. Sci. Res..

[58] Dada F.A., Nzewuji F.A., Esan A.M., Oyeleye S.I., Adegbola V.B. (2016). Phytochemical and antioxidant analysis of aqueous extracts of unripe pawpaw (*Carica papaya* Linn.) fruit’s peel and seed. Int. J. Recent Res. Appl. Stud..

[59] David H. (1983). The New Holistic Herbal.

[60] Singh P., Pandit S., Garnaes J., Tunjic S., Mokkapati V.R.S.S., Sultan A., Thygesen A., Mackevica A., Mateiu R.V., Daugaard A.E. (2018). Green synthesis of gold and silver nanoparticles from *Cannabis sativa* (industrial hemp) and their capacity for biofilm inhibition. Int. J. Nanomed..

[61] El-Deeb N.M., Khattab S.M., Abu-Youssef M.A., Badr A. (2022). Green synthesis of novel stable biogenic gold nanoparticles for breast cancer therapeutics via the induction of extrinsic and intrinsic pathways. Sci. Rep..

[62] Bahmanyar Z., Mohammadi F., Gholami A., Khoshneviszadeh M. (2023). Effect of different physical factors on the synthesis of spherical gold nanoparticles towards cost-effective biomedical applications. IET Nanobiotechnol..

[63] Mishra R.C., Kalra R., Dilawari R., Goel M., Barrow C.J. (2022). Bio-Synthesis of *Aspergillus terreus* Mediated Gold Nanoparticle: Antimicrobial, Antioxidant, Antifungal and In Vitro Cytotoxicity Studies. Materials.

[64] Sunkar S., Nachiyar V. (2013). Endophytes as potential nanofactories. Int. J. Chem. Environ. Biol. Sci.

[65] Abdel-Raouf N., Al-Enazi N.M., Ibraheem I.B. (2017). Green biosynthesis of gold nanoparticles using *Galaxaura elongata* and characterization of their antibacterial activity. Arab. J. Chem..

[66] Bawazeer S., Khan I., Rauf A., Aljohani A.S., Alhumaydhi F.A., Khalil A.A., Qureshi M.N., Ahmad L., Khan S.A. (2022). Black pepper (*Piper nigrum*) fruit-based gold nanoparticles (BP-AuNPs): Synthesis, characterization, biological activities, and catalytic applications–A green approach. Green Process. Synth..

[67] Kokila T., Ramesh P., Geetha D. (2016). Biosynthesis of AgNPs using Carica Papaya peel extract and evaluation of its antioxidant and antimicrobial activities. Ecotoxicol. Environ. Saf..

[68] Yang Z., Liu Z., Zhu J., Xu J., Pu Y., Bao Y. (2022). Green synthesis and characterization of gold nanoparticles from *Pholiota adiposa* and their anticancer effects on hepatic carcinoma. Drug Deliv..

[69] XRD Crystallite (Grain) Size Calculator (Scherrer Equation)-InstaNANO. https://instanano.com/all/characterization/xrd/crystallite-size/.

[70] Coates J. (2000). Interpretation of infrared spectra, a practical approach. Encycl. Anal. Chem..

[71] Aina A., Owolo O., Adeoye-Isijola M., Olukanni O., Lateef A., Egbe T., Aina F., Asafa T., Abbas S. Ecofriendly production of silver nanoparticles from the seeds of *Carica papaya* and its larvicidal and antibacterial efficacy against some selected bacterial pathogens. Proceedings of the IOP Conference Series: Materials Science and Engineering.

[72] Rahman A., Ismail A., Jumbianti D., Magdalena S., Sudrajat H. (2009). Synthesis of copper oxide nano particles by using *Phormidium cyanobacterium*. Indones. J. Chem..

[73] Phang Y.-K., Aminuzzaman M., Akhtaruzzaman M., Muhammad G., Ogawa S., Watanabe A., Tey L.-H. (2021). Green synthesis and characterization of CuO nanoparticles derived from papaya peel extract for the photocatalytic degradation of palm oil mill effluent (POME). Sustainability.

[74] Prasad C., Srinivasulu K., Venkateswarlu P. (2015). Catalytic reduction of 4-nitrophenol using biogenic silver nanoparticles derived from papaya (*Carica papaya*) peel extract. Ind. Chem..

[75] Kamaraj C., Karthi S., Reegan A.D., Balasubramani G., Ramkumar G., Kalaivani K., Zahir A.A., Deepak P., Senthil-Nathan S., Rahman M.M. (2022). Green synthesis of gold nanoparticles using *Gracilaria crassa* leaf extract and their ecotoxicological potential: Issues to be considered. Environ. Res..

[76] Singh H., Du J., Yi T.-H. (2017). Green and rapid synthesis of silver nanoparticles using Borago officinalis leaf extract: Anticancer and antibacterial activities. Artif. Cells Nanomed. Biotechnol..

[77] Sharma A., Sagar A., Rana J., Rani R. (2022). Green synthesis of silver nanoparticles and its antibacterial activity using fungus *Talaromyces purpureogenus* isolated from *Taxus baccata* Linn. Micro Nano Syst. Lett..

[78] Dheyab M.A., Aziz A.A., Jameel M.S., Khaniabadi P.M., Mehrdel B. (2021). Sonochemical-assisted synthesis of highly stable gold nanoparticles catalyst for decoloration of methylene blue dye. Inorg. Chem. Commun..

[79] Bhuiyan M.S.H., Miah M.Y., Paul S.C., Aka T.D., Saha O., Rahaman M.M., Sharif M.J.I., Habiba O., Ashaduzzaman M. (2020). Green synthesis of iron oxide nanoparticle using *Carica papaya* leaf extract: Application for photocatalytic degradation of remazol yellow RR dye and antibacterial activity. Heliyon.

[80] Hamelian M., Varmira K., Veisi H. (2018). Green synthesis and characterizations of gold nanoparticles using Thyme and survey cytotoxic effect, antibacterial and antioxidant potential. J. Photochem. Photobiol. B Biol..

[81] Ielo I., Rando G., Giacobello F., Sfameni S., Castellano A., Galletta M., Drommi D., Rosace G., Plutino M.R. (2021). Synthesis, Chemical-Physical Characterization, and Biomedical Applications of Functional Gold Nanoparticles: A Review. Molecules.

[82] Riaz T., Mughal P., Shahzadi T., Shahid S., Abbasi M.A. (2020). Green synthesis of silver nickel bimetallic nanoparticles using plant extract of *Salvadora persica* and evaluation of their various biological activities. Mater. Res. Express.

[83] Boomi P., Ganesan R., Poorani G.P., Jegatheeswaran S., Balakumar C., Prabu H.G., Anand K., Prabhu N.M., Jeyakanthan J., Saravanan M. (2020). Phyto-engineered gold nanoparticles (AuNPs) with potential antibacterial, antioxidant, and wound healing activities under in vitro and in vivo conditions. Int. J. Nanomed..

[84] Anadozie S.O., Adewale O.B., Fadaka A.O., Afolabi O.B., Roux S. (2022). Synthesis of gold nanoparticles using extract of *Carica papaya* fruit: Evaluation of its antioxidant properties and effect on colorectal and breast cancer cells. Biocatal. Agric. Biotechnol..

[85] Easmin S., Bhattacharyya M., Pal K., Das P., Sahu R., Nandi G., Dewanjee S., Paul P., Haydar M.S., Roy S. (2024). Papaya peel extract-mediated green synthesis of zinc oxide nanoparticles and determination of their antioxidant, antibacterial, and photocatalytic properties. Bioprocess Biosyst. Eng..

[86] Salla S., Sunkara R., Walker L.T., Verghese M. (2016). Antioxidant and apoptotic activity of papaya peel extracts in HepG2 cells. Food Nutr. Sci..

[87] D’Mello S.A., Finlay G.J., Baguley B.C., Askarian-Amiri M.E. (2016). Signaling pathways in melanogenesis. Int. J. Mol. Sci..

[88] Lorz L.R., Yoo B.C., Kim M.-Y., Cho J.Y. (2019). Anti-wrinkling and anti-melanogenic effect of *Pradosia mutisii* methanol extract. Int. J. Mol. Sci..

[89] Smit N., Vicanova J., Pavel S. (2009). The hunt for natural skin whitening agents. Int. J. Mol. Sci..

[90] Ullah S., Shah S.W.A., Qureshi M.T., Hussain Z., Ullah I., Kalsoom U.-E., Rahim F., Rahman S.S.U., Sultana N., Khan M.K. (2021). Antidiabetic and Hypolipidemic Potential of Green AgNPs against Diabetic Mice. ACS Appl. Bio Mater..

[91] Chinnasamy G., Chandrasekharan S., Bhatnagar S. (2019). Biosynthesis of silver nanoparticles from *Melia azedarach*: Enhancement of antibacterial, wound healing, antidiabetic and antioxidant activities. Int. J. Nanomed..

[92] Podsedek A., Majewska I., Redzynia M., Sosnowska D., Koziołkiewicz M. (2014). In vitro inhibitory effect on digestive enzymes and antioxidant potential of commonly consumed fruits. J. Agric. Food Chem..

[93] Solikhah T.I., Setiawan B., Ismukada D.R. (2020). Antidiabetic activity of papaya leaf extract (*Carica papaya* L.) isolated with maceration method in alloxan-induces diabetic mice. Syst. Rev. Pharm..

[94] Liu Y., Kim S., Kim Y.J., Perumalsamy H., Lee S., Hwang E., Yi T.-H. (2019). Green synthesis of gold nanoparticles using Euphrasia officinalisleaf extract to inhibit lipopolysaccharide-induced inflammation through NF-κB and JAK/STAT pathways in RAW 264.7 macrophages. Int. J. Nanomed..

[95] Phukan K., Devi R., Chowdhury D. (2022). Insights into Anti-Inflammatory Activity and Internalization Pathway of Onion Peel-Derived Gold Nano Bioconjugates in RAW 264.7 Macrophages. ACS Omega.

[96] Pandey S., Cabot P.J., Shaw P.N., Hewavitharana A.K. (2016). Anti-inflammatory and immunomodulatory properties of *Carica papaya*. J. Immunotoxicol..

[97] Pathak N., Khan S., Bhargava A., Raghuram G.V., Jain D., Panwar H., Samarth R.M., Jain S.K., Maudar K.K., Mishra D.K. (2014). Cancer Chemopreventive Effects of the Flavonoid-Rich Fraction Isolated from Papaya Seeds. Nutr. Cancer.

[98] John T., Parmar K.A., Kotval S.C., Jadhav J. (2021). Synthesis, characterization, antibacterial and anticancer properties of silver nanoparticles synthesized from carica papaya peel extract. Int. J. Nanosci. Nanotechnol..

[99] Agarwal R., Garg N., Kashyap S.R., Chauhan R. (2015). Antibacterial finish of textile using papaya peels derived silver nanoparticles. Indian J. Fibre Text. Res. (IJFTR).

[100] Sikkema J., de Bont J.A., Poolman B. (1994). Interactions of cyclic hydrocarbons with biological membranes. J. Biol. Chem..

[101] Rai A., Prabhune A., Perry C.C. (2010). Antibiotic mediated synthesis of gold nanoparticles with potent antimicrobial activity and their application in antimicrobial coatings. J. Mater. Chem..

[102] Sondi I., Salopek-Sondi B. (2004). Silver nanoparticles as antimicrobial agent: A case study on *E. coli* as a model for Gram-negative bacteria. J. Colloid Interface Sci..

[103] Velusamy P., Das J., Pachaiappan R., Vaseeharan B., Pandian K. (2015). Greener approach for synthesis of antibacterial silver nanoparticles using aqueous solution of neem gum (*Azadirachta indica* L.). Ind. Crops Prod..

[104] Cardillo D., Weiss M., Tehei M., Devers T., Rosenfeld A., Konstantinov K. (2016). Multifunctional Fe_2_O_3_/CeO_2_ nanocomposites for free radical scavenging ultraviolet protection. RSC Adv..

[105] Vasiljevic Z., Dojcinovic M., Vujancevic J., Jankovic-Castvan I., Ognjanovic M., Tadic N., Stojadinovic S., Brankovic G., Nikolic M. (2020). Photocatalytic degradation of methylene blue under natural sunlight using iron titanate nanoparticles prepared by a modified sol–gel method. R. Soc. Open Sci..

[106] Sorekine G., Anduwan G., Waimbo M.N., Osora H., Velusamy S., Kim S., Kim Y.S., Charles J. (2022). Photocatalytic studies of copper oxide nanostructures for the degradation of methylene blue under visible light. J. Mol. Struct..

[107] Chandrasekar A., Vasantharaj S., Jagadeesan N.L., Shankar S.N., Pannerselvam B., Bose V.G., Arumugam G., Shanmugavel M. (2021). Studies on phytomolecules mediated synthesis of copper oxide nanoparticles for biomedical and environmental applications. Biocatal. Agric. Biotechnol..

